# Neonatal onset Familial Mediterranean Fever

**DOI:** 10.1186/1546-0096-13-S1-P79

**Published:** 2015-09-28

**Authors:** ZB Özçakar, S Şahin-Kunt, S Özdel, F Yalçınkaya

**Affiliations:** 1 Ankara University, Pediatric Rheumatology, Ankara, Turkey

## Question

Familial Mediterranean fever (FMF) is an autosomal recessive disease, characterised by recurrent, self limited attacks of fever with serositis. Recently it was shown that FMF patients with early disease onset have more severe disease. The aim of our study was to describe the demographic, clinical and genetic features of FMF patients who had disease onset at the neonatal period.

## Methods

Files of patients who had been seen in our department (during routine follow-up visits) between January 2013 and January 2014 were retrospectively evaluated. Patients with disease onset during the neonatal period were included to the study.

## Results

Among 317 patients; 18 (7 females, 11 males) were enrolled. Consanguinity and family history of FMF were present in 28% and 56% of the patients, respectively. Clinical features seemed to be similar to general FMF patients; however, 50% of the patients were fussy children. The diagnosis of FMF was significantly delayed; the mean age at onset of therapy was 65.44 + 43.75 months. 38% of the patients had homozygous M694V mutation.

## Conclusions

Patients with FMF could have complaints even in the neonatal period. The smaller the age of disease onset, the more likely their diagnoses are delayed. Homozygous M694V mutation is a prominent mutation in this group of patients.

